# Habitat-specific foraging strategies in Australasian gannets

**DOI:** 10.1242/bio.018085

**Published:** 2016-06-15

**Authors:** Melanie R. Wells, Lauren P. Angel, John P. Y. Arnould

**Affiliations:** School of Life and Environmental Sciences (Burwood Campus), Deakin University, Geelong 3125, Australia

**Keywords:** Bio-logging, Camera, GPS, Foraging ecology, Local enhancement, Seabirds

## Abstract

Knowledge of top predator foraging adaptability is imperative for predicting their biological response to environmental variability. While seabirds have developed highly specialised techniques to locate prey, little is known about intraspecific variation in foraging strategies with many studies deriving information from uniform oceanic environments. Australasian gannets (*Morus serrator*) typically forage in continental shelf regions on small schooling prey. The present study used GPS and video data loggers to compare habitat-specific foraging strategies at two sites of contrasting oceanographic regimes (deep water near the continental shelf edge, *n*=23; shallow inshore embayment, *n*=26), in south-eastern Australia. Individuals from the continental shelf site exhibited pelagic foraging behaviours typical of gannet species, using local enhancement to locate and feed on small schooling fish; in contrast only 50% of the individuals from the inshore site foraged offshore, displaying the typical pelagic foraging strategy. The remainder adopted a strategy of searching sand banks in shallow inshore waters in the absence of conspecifics and other predators for large, single prey items. Furthermore, of the individuals foraging inshore, 93% were male, indicating that the inshore strategy may be sex-specific. Large inter-colony differences in Australasian gannets suggest strong plasticity in foraging behaviours, essential for adapting to environmental change.

## Abstract (precis)

**Summary:** Australasian gannets from two colonies in south-eastern Australia were equipped with GPS and miniature video data loggers to record foraging location and the prey preference.

## INTRODUCTION

Intraspecific variation in foraging behaviour occurs as a result of unpredictable resources and competition. Individuals within a population may specialise in specific behaviours in order to optimize their foraging efficiency and increase individual fitness (Bolnick et al., 2003; Sargeant, 2007); however little is known of the intrinsic factors influencing both the development and intensity of individual specialisations. Some factors suggested to influence the development of these specialisations are age, experience, sex, social status, and individual physical or physiological capacity (Schindler et al., 1997; Bearhop et al., 2006). The degree of intraspecific variation within a population indicates the species' plasticity – the ability to adapt to a change in environmental conditions by adopting specific behaviours (West-Eberhard, 1989; Grémillet and Charmantier, 2010; Samarra and Miller, 2015). Knowledge of intraspecific variation, therefore, is imperative in order to predict how populations may respond to future environmental variability.

The marine environment is highly spatio-temporally variable and predators have developed specialised foraging behaviours in order to locate and exploit unpredictable resources (Cherel and Hobson, 2007; Weimerskirch, 2007). While specialised foraging behaviour has been documented in many species (Bearhop et al., 2006; Woo et al., 2008; Ceia and Ramos, 2015), the exact means by which pelagic seabird species locate prey remains largely unknown (Weimerskirch, 2007; Sakamoto et al., 2009). Foraging efficiency may be increased by using conspicuous visual cues, such as predator aggregations, to locate prey patches (Thiebault et al., 2014a). This process, known as local enhancement (Thorpe, 1956), has led to many seabird species being observed feeding with conspecifics (other avian predators and marine mammals), often forming multispecies feeding associations (Au and Pitman, 1986; Harrison et al., 1991; Mills, 1998; Vaughn et al., 2007).

Members of the family Sulidae (gannets and boobies) employ a rapid aerial plunge-diving technique to hunt for small schooling prey (fish and cephalopods), utilising either quick V-shaped or longer U-shaped pursuit dives (Garthe et al., 2000; Ropert-Coudert et al., 2004; Machovsky-Capuska et al., 2011). Several studies have documented social foraging techniques utilised by gannets, such as local enhancement (Thiebault et al., 2014a,b; Tremblay et al., 2014), and revealed a degree of intraspecific and geographic variation in foraging strategies (Hamer et al., 2001; Grémillet et al., 2004; Garthe et al., 2007; Machovsky-Capuska et al., 2013a,b); however these studies have been confined to pelagic foraging habitats, limiting the current understanding of intraspecific variation within populations.

The Australasian gannet (*Morus serrator*) is a large pelagic seabird breeding on coastal locations and offshore islands along narrow continental shelves in south-eastern Australia and New Zealand. Its diet typically consists of small schooling prey such as pilchards (*Sardinops sagax*), anchovy (*Engraulis australis*), garfish (*Hyporhamphus melanochir*) and, to a lesser extent, larger species such as mackerel (*Trachurus declivis*), barracouta (*Thyrsites atun*), mullet (*Upeneichthys lineatus*) and squid species (*Nototodarus gouldi* and *N. sloanii*) (Bunce, 2001; Schuckard et al., 2012). Throughout its range it is an important top marine predator, with individuals from a single small colony in south-eastern Australia alone consuming an estimated 230 tonnes of fish and cephalopods during the breeding season (Bunce, 2001).

In Australia, gannet populations are increasing with new colonies becoming established (Norman et al., 1998; Pyk et al., 2013). While the underlying mechanisms for this increase are unknown, south-eastern Australian waters are among the fastest warming in the world and the region is likely to experience major oceanographic changes affecting the species' prey distribution (Lough and Hobday, 2011; Hobday et al., 2015). In central northern Bass Strait gannets nest on artificial structures scattered throughout Port Phillip Bay (Bunce et al., 2002), a shallow inshore embayment with an average depth of 14 m (Berelson et al., 1998). Little is known of the foraging strategies employed by gannets breeding in this environment and how individuals exploit the shallow waters of the bay (Angel et al., 2015a), and such knowledge is necessary to predict how this ecologically and economically (Parks Victoria, 2006) significant species may respond to the anticipated environmental changes. In contrast, birds breeding in western Bass Strait are located near a uniform continental shelf environment supported by a predictable annual nutrient-rich upwelling; hence these colonies are faced with contrasting conditions which allow for a comparative assessment of the behavioural strategies of foraging gannets.

Therefore, the aims of this study aims were to: (1) investigate the possible presence of unique foraging strategies of Australasian gannets; (2) compare foraging strategies between two sites of contrasting oceanographic regimes; and (3) assess differences in prey type relative to proximate foraging environment.

## RESULTS

**Fig. 1.**
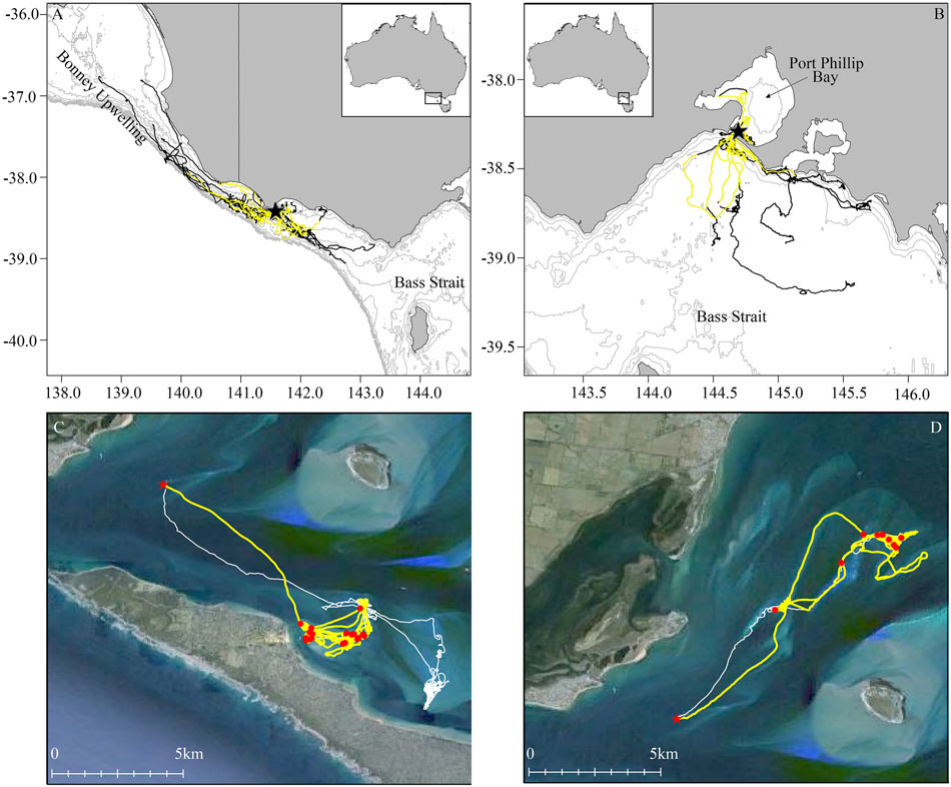
**Foraging tracks of individuals from Point Danger and Pope's Eye.** (A,B) GPS tracks of individuals from Point Danger (A, *n*=23) and Pope's Eye (B, *n*=26) indicated with black line and portion of foraging trip with video data available indicated with yellow line. Location of colonies indicated by black star. Bathymetry is represented at 20 m contours in light grey to continental shelf edge (200 m contour). (C,D) Representative GPS tracks (white line) overlaid on satellite imagery (Google Earth) of two birds from Pope's Eye (indicated by red star) displaying the inshore foraging strategy in shallow waters (C) and following sandbanks (D). Portion of the trip with video data indicated with yellow line and dive locations indicated with red circles.

A total of 49 individuals were instrumented (Point Danger: 11 males and 12 females; Pope's Eye: 17 males and 9 females). Birds from Point Danger were not significantly heavier in body mass (males: 2.51±0.05 kg; females: 2.69±0.07 kg) than birds from Pope's Eye (males: 2.44±0.1 kg; females: 2.58±0.05 kg) (Two-way ANOVA: *F*_1,62_=2.01, *P*=0.16). As such, the data for the two sites were combined, indicating females were significantly heavier than males (*F*_1,62_=5.95, *P*=0.02). The fine-scale GPS tracking data was obtained for 13.9±0.5 h at Point Danger and 12.3±1.0 h at Pope's Eye, although complete foraging trips were not recorded due to battery life limitations, these trips represented approximately 60% of the average total foraging trip duration (Angel et al., 2015a). All individuals from Point Danger foraged over the continental shelf in waters up to 100 m deep (bathymetric depth at dive locations: 43.4±2.1 m; Fig. 1A). Foraging in such deep waters, hereafter referred to as the pelagic strategy, is consistent with typical gannet foraging behaviour (Brothers et al., 1993; Grémillet et al., 2004; Garthe et al., 2007).

Conversely, only 50% (*n*=13) of instrumented individuals from Pope's Eye foraged in Bass Strait (bathymetric depth at dive locations: 49.9±1.9 m) and displayed the pelagic strategy (Fig. 1B). A high proportion of individuals who displayed the pelagic strategy were female, i.e. observed for 89% of females deployed (*n*=8) compared to 29% of males deployed (*n*=5). With the exception of one individual foraging in both Bass Strait and Port Phillip Bay, all individuals from Pope's Eye foraged within Port Phillip Bay. These individuals regularly had flight paths over shallow sand banks and nearshore habitats (bathymetric depth at dive locations: 8.9±0.9 m, Fig. 1C,D), this behaviour is hereafter referred to as the inshore strategy. In contrast to the pelagic strategy, the inshore strategy was predominantly undertaken by males (11 males and 1 female). No significant difference in body mass was found between males adopting the pelagic strategy (2.32±0.1 kg) or the inshore strategy (2.58±0.21 kg; *F*_1,6_=0.85, *P*=0.39).

To assess whether the two observed strategies influenced hunting behaviour, visual observations from the animal-borne video loggers were analysed. Due to device malfunction, simultaneous video data and GPS were obtained from 23 individuals at Point Danger and 20 individuals at Pope's Eye. Video capture lasted a mean of 3.4±0.2 h into the foraging trip as a result of battery constraints (range: 1.7-4.6 h).

From the 43 individuals for which video data was available, 467 dives were observed (10.9±1.6 dives per bird). Three birds did not perform dives during the video data period. The dive rate was similar between pelagic (3.6±0.7 dives h^−1^) and inshore strategies (4.2±0.7 dives h^−1^; *F*_1,38_=2.08, *P*=0.15), however the dive duration for birds adopting the pelagic strategy was significantly longer (14.9±1.2 s) than those adopting the inshore strategy (6.9±0.2 s; *F*_1,150_=59.4, *P*<0.001).

Merging of the video data with the GPS tracking data enabled the location of dives to be determined. No significant difference was observed in the spatial distribution of diving (pelagic strategy: 0.17±0.04 dives km^−1^; inshore strategy: 0.22±0.05 dives km^−1^) between the two strategies (*F*_1,38_=0.50, *P*=0.48); however the duration between subsequent dives was shorter for the pelagic strategy (0.13±0.02 h) than the inshore strategy (0.16±0.03 h; *F*_1419_=12.2, *P*<0.001), indicating that the pelagic strategy birds perform dives in rapid succession.

**Fig. 2.**
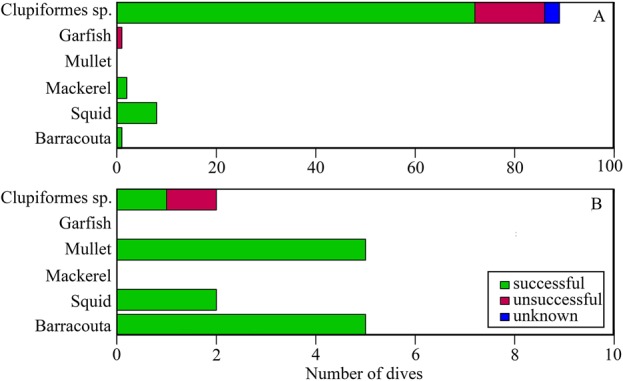
**Summary of prey targeted for all foraging dives made by Australasian gannets.** By using animal-borne video data loggers prey species were identifiable for both the pelagic (A) and inshore (B) foraging strategies. Prey capture of the targeted species was identified as either being successful (green), unsuccessful (purple) or unknown (blue).

**Fig. 3.**
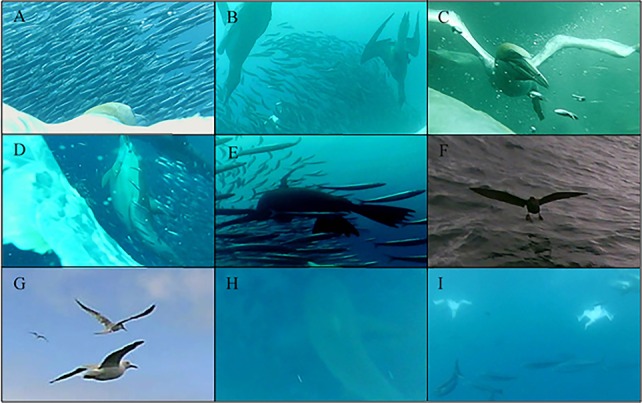
**Representive still images of typical pelagic foraging behaviour of Australaisan gannets.** Gannets feeding on small, schooling fish and bait balls (A,B), feeding with conspecifics (B,C), and other predators, such as dolphins (D), fur seals (E), shearwaters (F), terns and gulls (G), sharks (H) and tuna (I).

**Fig. 4.**
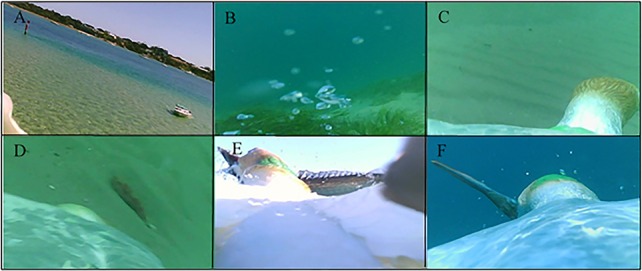
**Representative still images of behaviour and characteristics typical of inshore foraging strategy of Australasian gannets.** Gannets foraging alone (A), foraging in coastal shallow waters as evidenced by the seagrass (B) and sandy sea floor (C), and foraging on benthic (D) and large individual prey items (E,F).

Clear differences were observed in the prey targeted and capture success (Fig. 2). Birds adopting the pelagic strategy were observed to feed predominantly (89% of dives) on small, schooling fish (Clupeiformes spp., 25.2±2.9 g, Table 1; *n*=106 dives where prey was identifiable; Fig. 3A,B). In contrast, the inshore strategy individuals predominantly targeted large, non-schooling species (*n*=29 dives where prey was identifiable, Table 1) such as barracouta (33.5% of dives; 137.4±23.0 g), red mullet (33.5% of dives; 74.7±11.2 g) and garfish (7% of dives; 9.0±5.3 g; Fig. 4E,F). Interestingly, three individuals were observed to surface-plunge to capture demersal prey species (Fig. 4D). Inshore strategy individuals also targeted Clupeiformes spp., but to a lesser extent (13% of dives where prey was identifiable) than those using the pelagic strategy, and in none of these dives were inshore strategy individuals observed to be successful at capturing prey.

**Table 1.**
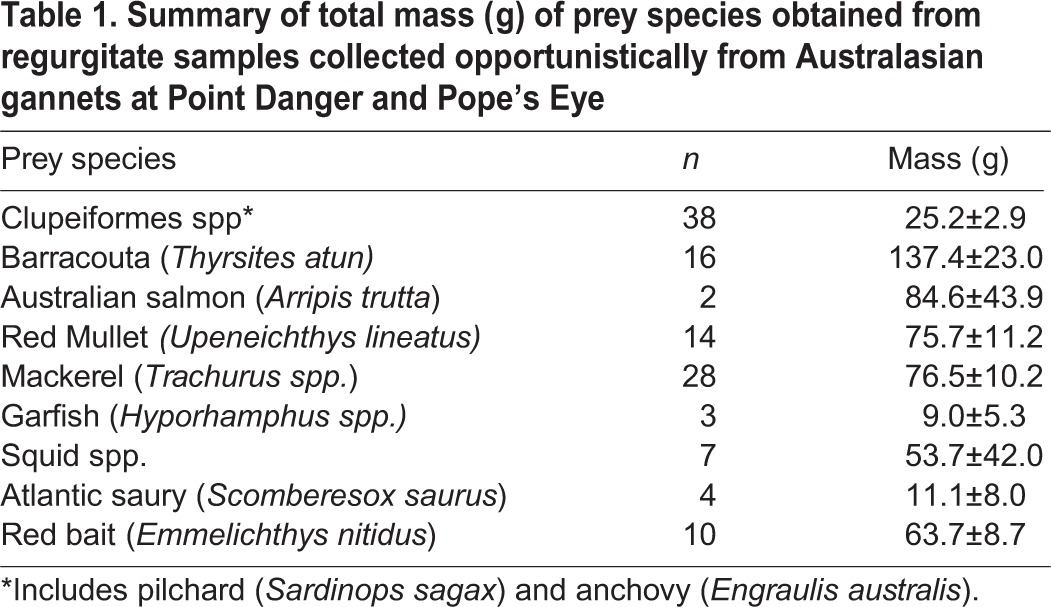
**Summary of total mass (g) of prey species obtained from regurgitate samples collected opportunistically from Australasian gannets at Point Danger and Pope's Eye**

The pelagic strategy individuals were noted to feed in multi-species feeding associations in 41% of the dives made, associating with conspecifics (Fig. 3B,C), dolphins (*Tursiops truncates, Delphinus delphis*; Fig. 3D) and Australian fur seals (*Arctocephalus pusillus doriferus*; Fig. 3E). Less frequently, species such as short-tailed shearwaters (*Puffinus tenuirostris*), terns (Sternidae spp.), gulls (Laridae spp.), little penguins (*Eudyptula minor*), sharks (Carcharhinidae spp.) and tuna (Scombrinae spp.) were observed foraging in these multi-species feeding associations (Fig. 3F-I). An exception to the general pattern of the pelagic strategy was observed in five dives from two individuals targeting larger prey, i.e. squid spp. (53.7±42.0 g) and mackerel (*Trachurus spp.*; 76.5±10.2 g, Table 1), when conspecifics and heterospecifics were absent. These individuals also foraged with conspecifics in subsequent dives within the same foraging trip. In contrast, individuals displaying the inshore foraging strategy exclusively foraged in the absence of conspecifics and heterospecifics (Fig. 4A) in shallow, coastal waters (Fig. 4B,C).

A total of 340 dives were recorded for the pelagic strategy where success could be determined (90.2% of total dives recorded), with 54.8±4.9% of dives (*n*=206) resulting in successful prey capture. Due to fewer individuals adopting the inshore strategy, only 79 dives were recorded where success could be determined (89.8% of total dives recorded) and in these, individuals were similarly successful with 66.2±8.6% of dives resulting in prey capture (*n*=59, *F*_1,38_=1.0, *P*=0.32).

## DISCUSSION

The combination of GPS and video data used in the present study revealed a degree of intraspecific variation in the foraging strategies of Australasian gannets. This variability indicates individuals can adapt their foraging behaviour to exploit contrasting environments, with birds foraging in pelagic waters feeding in multi-species feeding associations on small, schooling fish whereas birds exploiting shallow, benthic environments forage alone and on larger prey species. Differences in foraging strategies, particularly between benthic and pelagic foraging, have previously been based on movement and/or dive profiles in a range of seabird species (Grémillet et al., 1998; Tremblay and Cherel, 2000; Takahashi et al., 2003; Elliott et al., 2008). The present study provides support for the inferences of these studies by incorporating visual data to investigate behavioural and prey specific differences between strategies.

Gannet colonies are often located near the continental shelf edge due to increased productivity resulting in high prey availability in these areas. In the present study, gannets from the Point Danger colony, located near a highly productive upwelling (Butler et al., 2002), were found to forage in pelagic waters, as observed in previous studies on Cape, northern and Australasian gannets (Garthe et al., 2000; Machovsky-Capuska et al., 2011; Tremblay et al., 2014). However, the location of the Pope's Eye colony allows birds to forage in both pelagic and inshore environments resulting in the development of two foraging strategies within a single colony.

Perhaps as a consequence of the dimorphism observed between males and females (present study; Angel et al., 2015b), the inshore foraging strategy was predominantly adopted by males (with the exception of one female). Recent studies have found that in northern and Australasian gannets, as well as other Sulidae species, males forage closer inshore compared to females (the larger sex) which are observed to have greater range and trip duration (Lewis et al., 2002; Weimerskirch et al., 2006; Stauss et al., 2012; Cleasby et al., 2015). Additional hypotheses suggest territoriality (Matthews et al., 2008), parental roles or dietary requirements (Stauss et al., 2012) could also be the underlying mechanisms in the different strategies adopted by males and females strategy predominantly fed on schooling Clupeiformes species.

The diet of Australasian gannets has previously been well documented (Robertson, 1992; Schuckard et al., 2012; Tait et al., 2014), particularly at the Pope's Eye colony (Brothers et al., 1993; Norman and Menkhorst, 1995; Bunce and Norman, 2000; Bunce, 2001; T.M. Pyk, PhD thesis, Deakin University, Australia, 2012), with the majority of regurgitate samples comprised of schooling fish, barracouta and red mullet. These studies, however, have not explicitly linked diet with foraging location, therefore limiting the understanding of preferred prey. Norman and Menkhorst (1995) noted the considerable amount of barracouta recorded in regurgitate samples collected from breeding adults in Port Phillip Bay, concluding gannets prefer small schooling prey but opportunistically consume larger prey items. However, the results of the present study indicate birds foraging in shallow environments may preferentially target larger prey species, particularly when foraging along sand banks and shorelines (Fig. 1C,D).

In New Zealand, Australasian gannets display different dive behaviours and consume different prey species depending on colony location (Robertson, 1992; Schuckard et al., 2012; Machovsky-Capuska et al., 2013a,b). Similarly, northern gannets differ in foraging strategies and prey consumption in contrasting oceanographic environments, with individuals from an inshore colony feeding primarily on large prey species whereas birds from an offshore colony feed on small schooling prey (Garthe et al., 2007). Indeed, inter- and intra-individual variation in diet reflects the ability of species to fully exploit the available environment (Grémillet et al., 2004).

The video data in the present study also revealed the use of active wing flapping for submerged foraging without a preceding aerial plunge. Surface-plunging has been noted previously in both northern (Garthe et al., 2000) and Cape gannets (Ropert-Coudert et al., 2004), a behaviour associated with feeding on fishery discards on the sea surface or on schooling fish herded to the surface by multi-species feeding associations. However, the surface-plunges observed in the present study were used to capture demersal prey by inshore strategy individuals. This further highlights the adaptability of gannets in regard to the proximate environment and the prey available.

Seabirds may enhance their likelihood of finding food over short time scales by using public knowledge, either by travelling towards congregations of seabirds, (Local Enhancement hypothesis; Thorpe, 1956), or shadow the direction of departing and arriving birds to the breeding colony (Information Centre hypothesis; Ward and Zahavi, 1973), both of which may denote a profitable food patch. Alternatively, seabirds may possess private knowledge, using memory-based foraging route decisions to locate previously successful areas (Milinski, 1994). Northern gannets are thought to forage using both local enhancement and memory, with individuals displaying a high degree of memory-based decisions with alterations in their trips potentially due to local enhancement (Hamer et al., 2001; Pettex et al., 2012). As benthic environments are typically more predictable (Lalli and Parsons, 1993), the use of private knowledge would be beneficial for exploiting such habitats, whereas birds foraging in inconsistent, pelagic environments would more likely rely on public knowledge.

In the present study, birds adopting the pelagic strategy were accompanied by conspecifics when flying as has been observed in other gannet species (Thiebault et al., 2014a; Tremblay et al., 2014). Additionally, these individuals frequently foraged in multi-species feeding associations (Fig. 3B-I) where aquatic predators are likely to have aggregate prey near the sea surface (Fertl and Würsig, 1995) increasing prey capture success by gannets (Thiebault et al., 2016). In contrast, foraging in a shallow, predictable environment ensures prey are already within diving range and do not require other predators for it to be located. Correspondingly, individuals displaying the inshore strategy searched for prey alone along shallow sand banks and shorelines, potentially using in-prey silhouettes as hunting cues, with the sea floor often clearly visible during prey capture (Fig. 4B,C). Interestingly, prey capture success was similar between the two observed strategies suggesting inshore individuals have optimised their foraging efficiency. Assuming the dive rate recorded is indicative of an entire foraging trip, individuals of both strategies are employing a similar proportion of time obtaining prey; however as inshore strategy individuals are capturing larger prey items this could indicate these birds are more efficient in terms of a higher biomass consumed per unit time foraging.

In summary, the present study has revealed that Australasian gannets have the ability to adapt their foraging strategies to exploit resources and optimise foraging efficiency in different habitats. The strategy of foraging alone in an inshore environment and on demersal prey species has not been previously described in other gannet species, where individuals typically use local enhancement to locate small schooling fish. Furthermore, the inshore foraging strategy appears to be sex-specific, employed almost exclusively by males. Although similar sex-specific inshore behaviour is prevalent in other gannet species (Cleasby et al., 2015), explanations regarding the development and intensity of this specialisation remain largely unknown. Although only one foraging trip was recorded per individual in the present study, a concurrent study suggests individuals are faithful to their preferred strategy over multiple foraging trips (L.P. Angel, PhD thesis, Deakin University, Australia, 2015), further highlighting their ability to develop habitat-specific foraging strategies and exploit the prey available in various oceanographic regimes.

## MATERIALS AND METHODS

### Study sites and animal handling

The study was conducted during the incubation and chick rearing breeding stages of the 2014-15 breeding season (October-February) at Point Danger Coastal Reserve (38°23′36″S, 141°38′54″E) and Pope's Eye Marine Reserve (38°16′42″S, 144°41′48″E) (Fig. 1A,B). Point Danger (*ca* 660 pairs), a narrow continental shelf site, is located in western Bass Strait and is the site of Australia's only mainland gannet colony established in 1995 as overspill from a large colony (Lawrence Rocks, *ca* 3100 pairs) located 6 km offshore (Norman et al., 1998; Bunce et al., 2002). The colony is located in close proximity to the nutrient rich and highly productive Bonney Upwelling, south-eastern Australia's largest and most predictable upwelling (Nieblas et al., 2009) active during the Austral summer (November-April).

Pope's Eye (*ca* 180 pairs), established in 1985 also as overspill from Lawrence Rocks, is the largest of seven artificial structures hosting breeding gannets within Port Phillip Bay (Pyk et al., 2012). Port Phillip Bay is a shallow embayment with an average depth of 14 m (Berelson et al., 1998) comprised mostly of soft sandy sediments. It is located in northern Bass Strait, a shallow continental shelf region (average depth of 80 m) associated with highly mixed waters and relatively low nutrient input (Gibbs et al., 1986).

At both colonies, breeding adults were captured at the nest (by hand at Pope's Eye and with a noose-pole at Point Danger). To minimise disturbance, individuals that had previously been sitting on the nest were captured during a changeover between partners (Votier et al., 2013). Consequently, it was not logistically feasible to ensure a balanced sex ratio in the sampled animals. All animal handling followed protocols approved by Deakin University Animal Welfare Committee (A86/2010) and Department of Sustainability and Environment Victoria Wildlife Research (Permit 0005745). Individuals were weighed in a cloth bag using a spring scale (±25 g) and instrumented with a GPS data logger (IgotU120, Mobile Action Technology, Taipei, Taiwan, 44.5×28.5×13 mm, 20 g) and a miniature video data logger (Catnip Technologies Ltd., Hong Kong, 30×40×15 mm, 20 g; 400×400 pixels at 28-30 frames s^−1^). The GPS was positioned close to the preen gland and the video logger behind it, with the lens facing towards the head of the bird and slightly elevated at an angle of approximately 45° to maximise the field of view. Devices were packaged into a single unit with heat shrink tubing and attached to the central tail feathers using waterproof cloth tape (Tesa™ 4651, Hamburg, Germany). The devices and tape weighed <50 g, approximately 2% of body mass (*ca* 2.55±0.35 kg).

To observe fine-scale movements, GPS location was recorded (±10 m) every 10 s or every 5 s if velocity was >10 km h^−1^ (Thiebault et al., 2014b), while the video logger recorded continuously. Following device attachment, individuals were released close to the nest to resume normal behaviours with handling time lasting <10 min. Individuals were recaptured upon return to the colony after a single foraging trip, the devices removed, and a blood sample taken by venepuncture of the tarsal vein for genetic sexing (DNA Solutions, Wantirna, Australia).

### Data processing and analysis

GPS data was processed in the R statistical environment (R Core Team, 2015) using a speed filter (>80 km h^−1^) (McConnell et al., 1992) in the *diveMove* package (Luque, 2007). Video data were processed using VLC media player (VideoLan Organisation), with behaviour categorised visually at 1 s intervals. Behavioural information obtained from the video data was then overlaid on the foraging routes. The observed at-sea behaviours associated with foraging included: flying; diving (dive duration was determined from beginning of aerial descent until resurfacing on the water); and resting on the water (occurring in between dives). Additionally, the presence of conspecifics or heterospecifics was noted from the video data for each foraging trip. Bathymetric data were plotted in the R statistical environment at a 0.01° grid resolution and the values extracted for dive locations to determine average bathymetric depth for each foraging strategy.

When dives were detected in the video, they were analysed frame-by-frame and categorized as either: successful*,* if the individual captured prey, if the bill was open or if the gular pouch was enlarged upon resurfacing (Grémillet et al., 2006); unsuccessful, if the bill was visible during the entire dive and there was no evidence of prey capture; or success unknown, if neither the head nor bill could be seen and no prey capture observed. Where possible, prey was identified with the aid of reference collections and fish identification guides (Scott et al., 1974; Gomon et al., 1994).

Samples of prey species were collected opportunistically from birds handled during device recovery. Samples were placed in polyethylene bags and frozen until analysis. Prey items were identified to species level where possible and weighed (0.1 g), however due to partial digestion, accurate measures of prey length could not be obtained from all samples.

Assumptions for independence and normality of data were tested using a Chi-Square and Shapiro–Wilk's test, respectively. Where these assumptions were not met, data were log transformed to meet the assumptions. Unless otherwise indicated, results are presented as mean±standard error (s.e.m.).
